# THE ROLE OF HOUSING MANAGEMENT AND STAFF IN ADMINISTERING THE R3 PROGRAM

**DOI:** 10.1093/geroni/igz038.2854

**Published:** 2019-11-08

**Authors:** Elizabeth Simpson, Edward Miller, Pamela Nadash, Taylor Jansen, Yan Lin, Marc Cohen

**Affiliations:** University of Massachusetts Boston, Boston, Massachusetts, United States

## Abstract

Existing staff and management are central players in integrating enhanced services into affordable senior housing. This study describes the experience of housing providers in the implementation and operations of the R3 program. Semi-structured interviews were conducted with executives and direct service staff across the four intervention sites. Results indicate that staff served an important role in facilitating resident recruitment by operating as trusted sources of information about the R3 program. Top-level support for R3, acculturating R3 staff to the housing site, developing communication and data systems, and integrating new and existing staff were seen as crucial to the success of the program. Benefits noted by housing staff included freedom to redirect one’s energies/focus, production of actionable data/insights, reductions in resident turnover, and the addition of a nurse to the onsite services team. Housing management/staff experience with R3 can serve as a guide to moving to an enhanced services model.

